# Tracking bacteria at high density with FAST, the Feature-Assisted Segmenter/Tracker

**DOI:** 10.1371/journal.pcbi.1011524

**Published:** 2023-10-09

**Authors:** Oliver J. Meacock, William M. Durham

**Affiliations:** 1 Department of Biology, University of Oxford, Oxford, United Kingdom; 2 Department of Physics and Astronomy, University of Sheffield, Sheffield, United Kingdom; 3 Department of Fundamental Microbiology, University of Lausanne, Lausanne, Switzerland; Georg-August-Universitat Gottingen, GERMANY

## Abstract

Most bacteria live attached to surfaces in densely-packed communities. While new experimental and imaging techniques are beginning to provide a window on the complex processes that play out in these communities, resolving the behaviour of individual cells through time and space remains a major challenge. Although a number of different software solutions have been developed to track microorganisms, these typically require users either to tune a large number of parameters or to groundtruth a large volume of imaging data to train a deep learning model—both manual processes which can be very time consuming for novel experiments. To overcome these limitations, we have developed FAST, the Feature-Assisted Segmenter/Tracker, which uses unsupervised machine learning to optimise tracking while maintaining ease of use. Our approach, rooted in information theory, largely eliminates the need for users to iteratively adjust parameters manually and make qualitative assessments of the resulting cell trajectories. Instead, FAST measures multiple distinguishing ‘features’ for each cell and then autonomously quantifies the amount of unique information each feature provides. We then use these measurements to determine how data from different features should be combined to minimize tracking errors. Comparing our algorithm with a naïve approach that uses cell position alone revealed that FAST produced 4 to 10 fold fewer tracking errors. The modular design of FAST combines our novel tracking method with tools for segmentation, extensive data visualisation, lineage assignment, and manual track correction. It is also highly extensible, allowing users to extract custom information from images and seamlessly integrate it into downstream analyses. FAST therefore enables high-throughput, data-rich analyses with minimal user input. It has been released for use either in Matlab or as a compiled stand-alone application, and is available at https://bit.ly/3vovDHn, along with extensive tutorials and detailed documentation.

## Author summary

Much of what we know about bacterial behaviour comes from tracking solitary cells through space and time. For example, one can unpick the mechanisms that drive chemotaxis by quantifying the movement of individual microbes as they respond to a nutrient source. However, in infections, industrial processes and the environment, bacteria usually live in tightly-packed communities where they display unique behaviours not observed in solitary cells, such as the activation of contact-dependent weapons to kill their neighbours. Tracking individuals in these dense assemblages is technically challenging because it is difficult to follow cells using only their position in each frame. Here we present a new software tool called FAST which combines machine learning and information theory to optimise cell tracking. Our approach uses a range of different cell characteristics such as shape and fluorescent intensity to better distinguish individuals over time, reducing errors up to 10-fold compared to traditional approaches. In addition, FAST estimates how tracking accuracy changes over time, alerting users to potential problems such as out of focus frames. By tracking cells in a diverse range of conditions with minimal user input, FAST provides a new quantitative platform to study how bacteria have adapted to live in groups.

## Introduction

**Fig 1:**
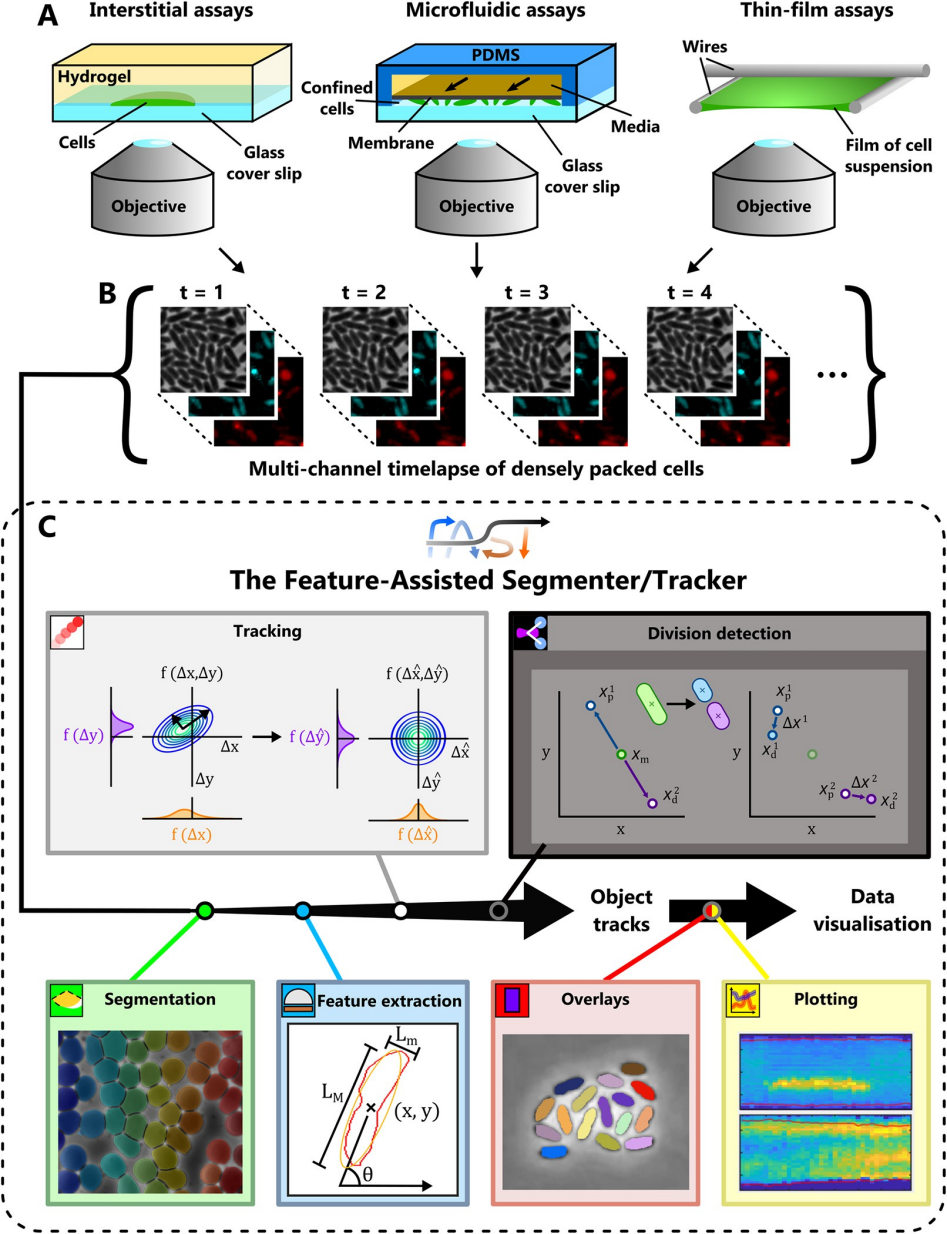
Analysis of high-density datasets using FAST’s modular framework. (A) While bacteria naturally form monolayers in some environments, a number of different assays are used to physically confine cells to a plane in laboratory experiments. Among these are interstitial colonies, microfluidic devices that trap cells between two solid boundaries, and experimental assays that confine cells to a thin film of liquid. (B) These experiments are typically imaged using automated microscopy systems capable of collecting images in both transmitted light and fluorescent channels at specified time points. (C) FAST analyses these imaging datasets in six separate modules, each of which is used in sequence (see S1 Text). A complete specification of each module is provided in section 3 of S1 Text.

Time-lapse microscopy and automated cell tracking has led to many fundamental advances in our understanding of how microorganisms sense and respond to their environment. While many studies have focused on the movement of planktonic bacteria at relatively low densities, many behaviours—including collective movement [1,2], combat [3], sharing of public goods [4] and genetic exchange [5]—typically only occur in the closely-packed assemblages in which most microbes live [6,7]. These dense communities are often studied in the laboratory using confluent monolayers of cells, which are much easier to image than three-dimensional aggregations. One method to generate such monolayers is to confine cells with a slab of agarose or polyacrylamide to form an interstitial colony [1,8–12], while more advanced microfluidic techniques [13] can also be used to confine cells to a single plane while allowing for more precise control over their chemical environment (Fig 1A). Monolayers can also form in thin films of fluid, including those arising naturally during bacterial swarming motility [2] and in assays used to study mixing induced by flagellar motility [14]. Regardless of the origin of the monolayer however, investigators face the same technical challenges when tracking densely packed cells using phase-contrast, brightfield and/or epi-fluorescence microscopy (Fig 1B).

Tracking solitary cells at low density is relatively straightforward, and basic tracking algorithms that use cell position alone often produce excellent results. However, tracking cells that are densely packed together is notoriously difficult because the spacing between neighbouring cells becomes similar to the distance cells move between frames. These problems are exacerbated by cell motility. While some species of bacteria are non-motile at high density and spread slowly only via cell division and the secretion of surfactants [9,13], emergent patterns of collective motility driven by twitching [1], swarming [2] and gliding [15] further complicate tracking by rapidly changing the positions of cells. Consequently, dense, motile communities must be imaged at high framerates for tracking to be feasible, such that a typical experiment requires a timeseries of hundreds or thousands of images. The large size of imaging datasets, combined with the large number of cells within each image, means that the computational time required for cell tracking is often one of the main bottlenecks in a researcher’s workflow. This can be compounded if tracking must be repeated multiple times to optimise tracking parameters.

A basic nearest-neighbour tracking algorithm compares the coordinates of cell centroids between subsequent frames and builds trajectories by connecting those centroids that are closest together between subsequent time points. More advanced algorithms leverage additional cell characteristics or ‘features’ to distinguish cells from one another, including metrics that measure cell shape, orientation, fluorescence levels and patterns of previous movement [16–19]. However, incorporating these additional feature measurements introduces a new problem, namely how to efficiently combine the information from different features to optimise tracking performance. In some software packages (e.g. MicrobeJ [17]), this requires the user to manually choose a large number of parameters and make qualitative judgements of the trajectories that result from each set of parameters. The combinatorial explosion in the number of possible parameters, the fact that a single parameter set can require hours to test, and the lack of a rigorous way to compare tracking results across different parameter sets, means that parameter optimisation in such a tracking algorithm is typically a highly iterative, time-consuming process.

A related problem is the fact that properties of bacteria within collectives are highly dynamic, changing at both the individual level and population level over time. For example, fluorophores can accumulate within cells or bleach, while cell movement can speed up or slow down due to the secretion of extracellular factors or changes in gene expression [20,21]. In addition, the overall density of cells often increases over time as a result of cell division. This variability means that an algorithm optimised to track cells at the beginning of an experiment might struggle at later timepoints. Furthermore, experimental issues—such as changes in illumination, focus, or shifts in the field of view caused by thermal drift—can cause an abrupt deterioration of tracking accuracy. Knowing when tracking accuracy has deteriorated to an unacceptable level often lacks a rigorous basis and requires the output of tracking software to be carefully validated by eye, which is typically infeasible for high-throughput datasets.

In this paper, we discuss a new approach that uses unsupervised machine learning to improve the fidelity of tracking. Our system automatically measures the statistical properties of each feature over time and then uses this data to dynamically change the relative weighting of each feature based on the information it can contribute to solving the tracking problem. It also provides users with a metric of the expected accuracy of the resulting cell trajectories, alerting them to sections of datasets that may need to be omitted from subsequent analyses. This tracking algorithm is combined with robust segmentation, feature extraction, lineage analysis and visualisation routines to make up FAST, the Feature-Assisted Segmenter/Tracker. FAST has been released as open-source software which can be run either directly within Matlab or as a stand-alone application, and has already been used in a number of publications to accurately analyse densely-packed bacterial monolayers [1,10,22,23].

In the following sections, we discuss the design approach and general structure of FAST, and then illustrate the utility of our novel cell tracking approach using synthetic datasets. Next, we discuss three case studies that illustrate the versatility of FAST, including: 1) lineage analysis of *E*. *coli* microcolonies, 2) tracking of twitching *P*. *aeruginosa* cells in a 2D monolayer, and 3) automated analysis of the Type 6 Secretion System (T6SS) in a co-culture of *P*. *aeruginosa* and *V*. *cholerae*. While these case studies focus on densely packed bacteria, FAST can also be used to analyse other types of biological samples (*e*.*g*. Fig Ag-i in S1 Text).

## Results

### Software overview

Initially, we conducted a review of existing cell tracking software packages [17,24–28] to establish four key design objectives for our software: modularity, rapid user feedbacks, minimisation of user-defined parameters and extensibility (see section 2 of S1 Text for further details). We built the FAST pipeline following these design principles, resulting in a set of six modules that are used in sequence (Fig 1C). If required, external tools can be used to pre-process timelapse images—for example, to stabilise or bleach correct images—before importing them into FAST. Imaging data is loaded into FAST using Bioformats [29], ensuring compatibility with a diverse range of imaging formats. The first module of FAST is the Segmentation module, which uses standard image processing methods—including texture detection, ridge detection [30] and a watershed algorithm [31]—to identify the boundaries of individual cells. While this is typically based on brightfield or phase-contrast images, fluorescent images can also be used if cells are appropriately labelled with cytosolic or membrane-bound fluorophores. Next, the Feature Extraction module measures a range of different cell properties such as position, size and fluorescent intensity (potentially in multiple channels) using the previously extracted segmentation as a basis. The Tracking module employs our machine learning process to quantify the information associated with each feature and then calculates the relative weighting of each feature to maximise tracking fidelity. If required, a manual validation and correction sub-module also allows the user to correct any mistakes made by the tracking algorithm. The optional Division Detection module uses a closely related machine learning process to assign daughter cells to mother cells following cell division events. Two separate modules can finally be used to visualise the output of FAST: the Overlay module plots trajectories and/or the results of analyses over the top of the original images, while the Plotting module contains a range of different options to visualise extracted data. A comprehensive description of each of these modules is provided in section 3 of S1 Text.

The FAST GUI guides the user through the process of analysing a single dataset. However, many applications require a large number of imaging datasets to be analysed using consistent settings. To automate this, we have implemented a batch-processing tool called doubleFAST. Once a user has performed an analysis on a single dataset using the FAST GUI, doubleFAST can then read the settings used during this initial run and automatically apply them to any number of additional datasets. This allows data from multiple experiments to be processed with minimal amounts of additional user input and ensures each has been analysed using consistent settings.

Finally, we have implemented a post-processing toolbox, which contains scripts and functions to perform a number of different tasks on FAST’s output. These allow, among other things, conversion of track data to other file formats, automated detection of different genotypes, and the annotation of events such as reversals in movement direction. Users of FAST can suggest new additions to this toolbox so they can be used by the wider community.

### FAST’s tracking algorithm

One of FAST’s principal innovations is its tracking algorithm, which automatically determines how to best combine data from a variety of different cell features to improve tracking fidelity. Although previous tracking software packages have had the option to incorporate cell characteristics other than position in their tracking routines [16,19], FAST uses a conceptual framework based on information theory that optimises this process, increasing both the power and convenience of this approach. In this section we provide a high-level overview of our main innovations and how they impact the tracking process. For more detailed derivations and explanations, please refer to section 3.4 of S1 Text.

Our approach to the tracking problem is based on measurement of the amount of information that can be used to assign links between objects in subsequent frames, a measurement we call the ‘trackability’. This trackability score provides an integrated measure of how accurately we can follow objects from frame to frame, and due to its grounding in information theory has certain desirable properties such as the additivity of contributions from statistically independent features [32,33]. Measuring the trackability over time therefore provides users with a tool to predict when tracking will be stable and robust, as well as flagging portions of a dataset that might yield spurious trajectories. We define trackability in the following paragraphs and then illustrate how this metric is used in practice in our first and second case studies.

We begin by assuming that *N* different features are measured for each object. An object’s features can be expressed as a vector $x_{t}^{i}$ with *N* elements, where the object index is denoted as *i* and time is denoted as *t*. Changes in an object’s features over time—corresponding to, for example, translational movement or changes in fluorescent intensity—therefore generate a trajectory through the *N-*dimensional feature space. The goal of our tracking algorithm is to reconstruct each object’s trajectory from the cloud of individual data points resulting from the segmentation and measurement of objects in each image. As previously noted, individuals in high-density and high-motility systems can easily move further than the typical cell-cell separation between frames, making it difficult to reconstruct their trajectories from positional information alone. By including additional features in the tracking framework, we expand the feature space from these two spatial dimensions to *N* feature dimensions, creating new axes along which one can potentially discriminate neighbouring individuals from each other.

The trackability metric provides an estimate of how distinguishable trajectories are from each other in the feature space, and therefore how accurate tracking is likely to be. Unpredictable movement of objects tends to reduce trackability, while trackability increases if the features sample a wider range of values (*i*.*e*. if they have a larger dynamic range). To formalise this, we model an object’s instantaneous position in feature space as the random vector ***X***_*t*_ and the change in this position between subsequent images as the random vector Δ***X***_*t*_. The distribution *f*(***x***) is then the probability density function (PDF) representing the chance of finding a randomly selected object at a particular position ***x*** in the absence of additional information (specifically, the position of the object at prior timepoints), while the distribution *f*(Δ***x***) represents the stochastic change in an object’s position in feature space between sequential timepoints. We estimate *f*(Δ***x***) by assuming that the motion of an object through feature space can be modelled as a Gaussian random walk. Explicitly, we assume that the feature vector $x_{t+1}^{i}$ of an object at frame *t*+1 can be written in terms of its prior feature vector $x_{t}^{i}$ as:

$$x_{t+1}^{i}=x_{t}^{i}+{\Delta X}_{t},$$ (1)

where Δ***X***_*t*_ is modelled as a multivariate normal $N(\mu_{t}(\Delta x),\Sigma_{t}(\Delta x))$, and ***μ***_*t*_(Δ***x***) and Σ_*t*_(Δ***x***) are respectively the mean vector and covariance matrix of the set of frame-frame feature displacements {Δ***x***_*t*_}. We can similarly characterize *f*(***x***) using the covariance matrix of the raw object locations, Σ_*t*_(***x***). While we can estimate Σ_*t*_(***x***) directly from static snapshots as the covariance of the set of feature vectors {***x***_*t*_}, resolving {Δ***x***_*t*_} and subsequently ***μ***_*t*_(Δ***x***) and Σ_*t*_(Δ***x***) requires a putative set of cell trajectories, which in our algorithm are obtained via a preliminary round of tracking that uses a simple nearest-neighbour approach. To help ensure these summary statistics are measured accurately, FAST uses only the lowest displacement trajectory links from this training dataset, which are the ones most likely to be correct. Users specify the fraction *F* of the links with the smallest displacements to be used in the calculation of ***μ***_*t*_(Δ***x***) and Σ_*t*_(Δ***x***). This allows the user to balance the trade-off between larger values of *F*, which increases the size of the training dataset, and smaller values of *F* which increases the quality of the training dataset.

From these measurements, we estimate the amount of information available for assigning objects between frames by calculating the difference between the entropies for the two distributions, H(***X***) and H(Δ***X***) [33,34]. The trackability *r*_*t*_, which is measured in bits/object, can then be written as:

$$r_{t}=\frac{1}{2}{log}_{2}\left(\frac{\left|\Sigma_{t}(x)\right|}{\left|\Sigma_{t}(\Delta x)\right|}\right)+\frac{N}{2}{log}_{2}\left(\frac{6}{\pi e}\right)-{log}_{2}(n_{o,t}),$$ (2)

where *n*_*o*,*t*_ is the total number of objects present at time *t* and |∙| denotes the determinant of the contents. The first term captures the balance between the dynamic range of all features (|Σ_*t*_(Δ***x***)|) and the unpredictability of an object’s movement through feature space (|Σ_*t*_(Δ***x***)|), while the second and third terms rescale this quantity to express the amount of information available to the tracking algorithm per object. For a further details, including our assumptions about the form of the underlying statistical distributions, please see section 3.4.2 of S1 Text.

**Fig 2:**
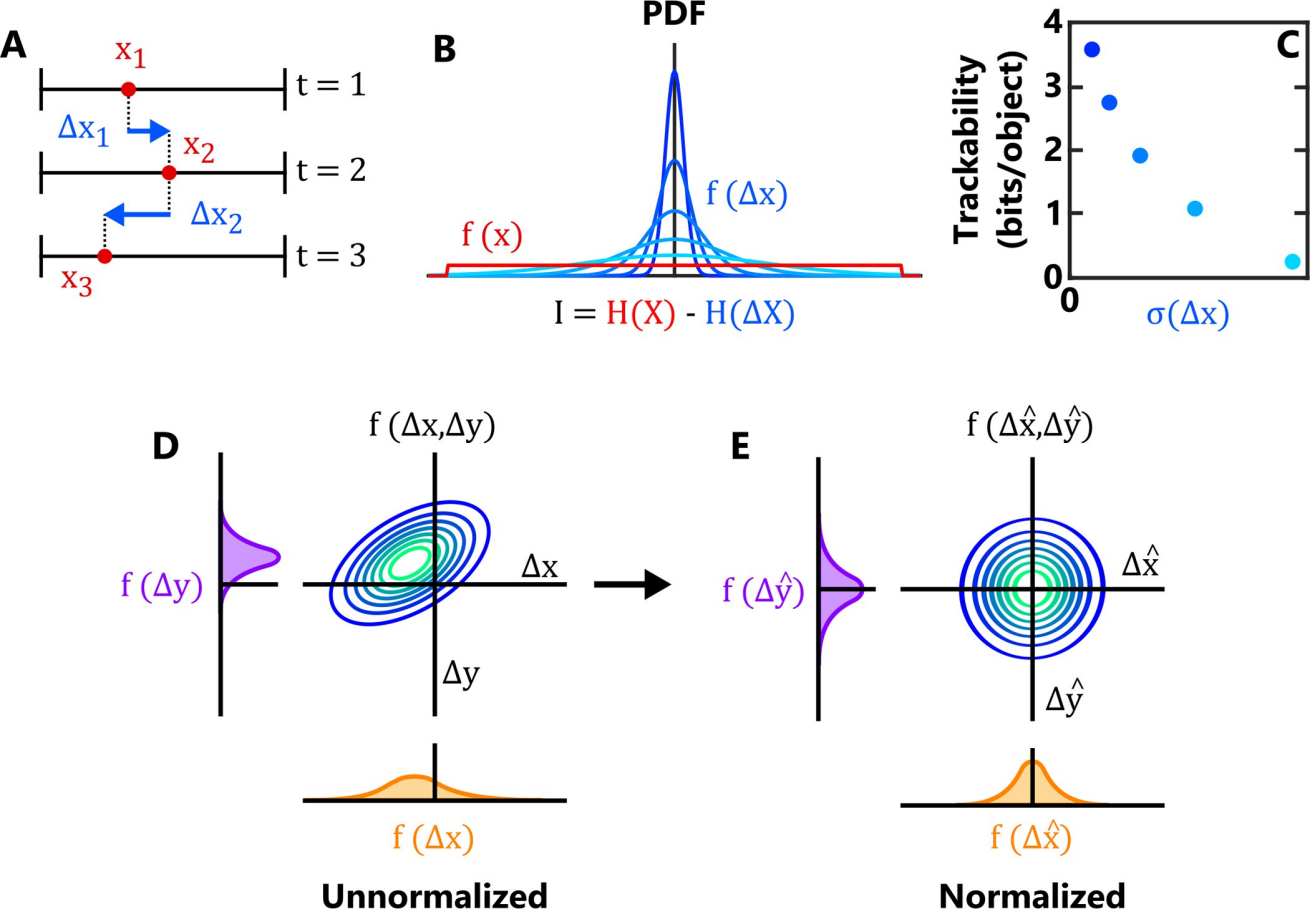
An information-theoretic framework for automated object tracking. (A) For illustrative purposes, we consider here a theoretical dataset in which an object is characterised using a single feature, its position along the *x*-axis. The object’s position at three successive timepoints is denoted *x*_1_, *x*_2_, *x*_3_ (red circles), while the displacements are denoted Δ*x*_1_, Δ*x*_2_ (blue arrows). (B) We assume that feature displacements are drawn from a Normal distribution *f*(Δ*x*), while the instantaneous object position (independent of knowledge of other timepoints) is drawn from a separate Uniform distribution *f*(*x*). The information content *I* of the feature is then calculated as the difference in the entropies of the two distributions, H(*X*) and H(Δ*X*), and represents our increase in certainty about the position of the object at time *t*+1 given knowledge about its position at time *t*. The trackability quantifies the total amount of information measured for each object, which increases when *f*(Δ*x*) exhibits less variability relative to *f*(*x*). (C) Trackability decreases when the distribution of *f*(Δ*x*) is broader (i.e. the feature becomes more ‘noisy’). Here the different colours correspond to the different distributions of *f*(Δ*x*) shown in panel B. (D,E) Illustration of the feature normalization process for two features. In both D and E, the central plot indicates the joint distribution of a pair of feature displacements, while the left and bottom plots indicate the corresponding marginal distributions. In D, the random variables representing the unnormalized frame-frame displacements of the two features—Δ*X* and Δ*Y*—are correlated and displaced from the origin. Using the joint distribution’s covariance matrix *Σ*(Δ***x***) and mean vector ***μ***(Δ***x***), the feature space is transformed such that the resulting joint distribution of feature displacements $f(\Delta \hat{x},\Delta \hat{y})$ is zero-centred and isotropic (E), ensuring that each feature exhibits an equivalent amount of stochastic variation between frames.

To illustrate the trackability metric, we consider the case of a single object with a single feature, for example its position in space along a single axis, *x* (Fig 2A–2C). This simplifies Eq (2) to:

$$r_{t}=\frac{1}{2}{log}_{2}\left(\frac{{\sigma_{t}(x)}^{2}}{{\sigma_{t}(\Delta x)}^{2}}\right)+\frac{1}{2}{log}_{2}\left(\frac{6}{\pi e}\right),$$ (3)

where *σ*_*t*_(*x*) and *σ*_*t*_(Δ*x*) denote the standard deviation of their respective distributions. As expected, one observes a larger trackability score when the distribution of feature displacements, *f*(Δ*x*), is more sharply peaked relative to the distribution of *f*(*x*) (Fig 2B and 2C). *i*.*e*. when the typical distance an object moves between frames is small compared to the range of values of *x*. Intuitively, the precision of tracking will increase as (*i*) the size of the random fluctuations in feature space decreases, (*ii*) the number of objects within a frame decreases, and (*iii*) the total size of the feature space the objects occupy (*i*.*e*. their dynamic ranges) increases. By taking these multiple factors into account, *r*_*t*_ represents an integrated measurement of the risk that a given object will be incorrectly linked to a different object in a subsequent frame.

In addition to calculating the trackability, ***μ***_*t*_(Δ***x***) and Σ_*t*_(Δ***x***) are also used to perform what we call ‘feature normalization’. This transformation converts the raw feature space ***x*** to a normalized feature space $\hat{x}$ with a corresponding displacement distribution $f(\Delta \hat{x})$ that is isotropic and zero-centred (Fig 2D and 2E), thus ensuring that the stochastic variation observed within each component of $\hat{x}$ is equal and that any predictable motion in the feature space (e.g. a gradual increase in cell length due to growth, or a reduction in fluorescent intensity due to photobleaching) is accounted for. The metric of this transformed space is the Mahalanobis distance, a dimensionless measure of how reliably we can predict where an object will appear in the feature space at the next time point under the assumptions of our statistical framework. By providing an equitable way to combine data from different features together, this metric allows us to more accurately distinguish correct from incorrect putative links. Large distances between sequential timepoints in this space indicate a discrepancy between the predicted and observed location of an object in the next frame, and so suggest that the input data must be erroneous—for example, because an object was mis-segmented in a single frame. In contrast to previous approaches where feature weightings have to be selected or measured manually [16], feature normalization allows one to automatically optimise the contribution of multiple features without any additional user input.

Finally, we use these statistical measurements to also calculate the adaptive tracking threshold *β*_*t*_, which automatically adjusts the stringency of the algorithm that links objects together based on how much information is available in each frame. While a larger fraction of putative links are accepted when information is relatively plentiful, only the highest certainty links are accepted when information is more limited. This dynamic adjustment of the link threshold thus allows FAST to maximise the number of trajectories in less challenging tracking conditions (*e*.*g*. slow-moving cells at low density) while minimizing the number of spurious links in more challenging conditions (*e*.*g*. fast-moving cells at high density). As different imaging datasets may require a different balance between maximising the number of correct links and minimising the number of spurious links, users select a static threshold *P* to balance this trade-off, which FAST then automatically converts to the time-varying threshold *β*_*t*_ based on the instantaneous amount of information available.

In summary, users must specify the tracking threshold *P*, the fraction of training links to retain *F* and the features that should be included in the final round of tracking. To help users optimise these parameters quickly, the Tracking module contains built-in tools that allow users to objectively assess tracking quality on a single pair of frames before committing to processing the entire dataset.

### Validating the methodology of FAST using ground-truthed datasets

**Fig 3:**
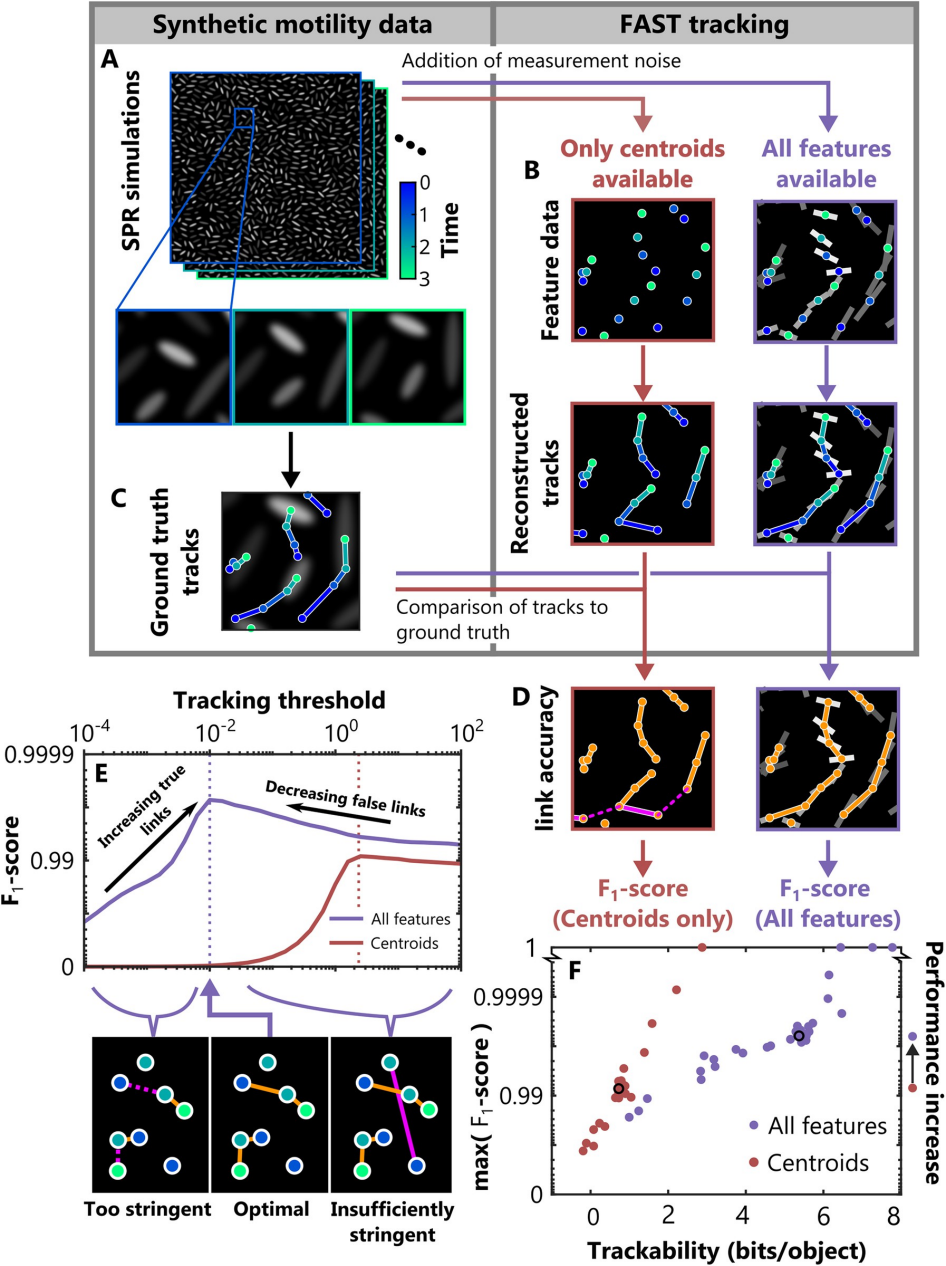
Validating FAST using synthetic data from a simulation of collective bacterial motility. (A) We used a self-propelled rod (SPR) model to generate noisy datasets with a verified ground truth. We then performed cell tracking with FAST using either only a single feature (the rod’s centroid at sequential timepoints; brown) or using a suite of different features (rod centroid, length, orientation and simulated fluorescent intensity; purple). Comparison of the reconstructed trajectories from FAST (B) with the ground truth data (C) allowed us to identify the errors made by the tracking algorithm (D). Errors were split into two categories: links made between objects that were incorrect (‘false positives’, solid magenta lines), and links between objects that were missed (‘false negatives’, dashed magenta lines). We also calculated the number of correct links made in each case (‘true positives’, solid orange lines). From these counts, we evaluated the performance of the tracking algorithm by calculating the *F*_1_-score (main text). (E) The value of this *F*_1_-score depends on a user-defined parameter, the tracking threshold. To objectively compare the results from the tracking algorithm when run on datasets with different properties, we calculated the tracking threshold that generated the largest *F*_1_-score for a given dataset and used this score in subsequent analyses. (F) Including all feature information substantially and consistently improved tracking performance compared to when only object positions were used (see also S2 Fig). The arrow to the right of the plot (labelled ‘Performance increase’) indicates the median *F*_1_-score for the two cases, corresponding to a ~10-fold reduction of tracking errors when all features were used. Furthermore, we found that our trackability metric was an excellent predictor of tracking accuracy for a given set of features, suggesting that it can be used to estimate the accuracy of the algorithm even when a ground truth is not available. In the different simulations we varied rod density, propulsive force, and framerate, as well the amount of noise in the measurement of position, length and fluorescence (Table 1). The black circles in F correspond to the dataset presented in E.

To demonstrate the functionality of our tracking algorithm and to illustrate how using multiple features can enhance accuracy, we used a previously described self-propelled rod (SPR) model [1,35] to generate synthetic datasets that simulate bacteria collectively moving at high-density (Fig 3A). In these simulations, cells are modelled as stiff, mutually repulsive rods that are propelled by a constant force (see Methods). This model has been shown to closely approximate bacterial collectives that propel themselves using either flagella or type IV pili, where steric interactions between neighbouring cells generate complex emergent collective behaviours [1,35]. Importantly, this approach allows us to independently control each of the different properties of the system, allowing us to both test FAST on a very large number of qualitatively different datasets and to objectively assess its performance using an automatically generated ground truth.

We simulated a 2D monolayer of cells constitutively expressing a fluorescent protein by initialising populations of cells whose length and fluorescent intensity were drawn from distributions obtained from experimental data (S1 Fig). Following initialization, we simulated cell movement by numerically integrating the equations of motion for each rod. Once the system had reached steady-state, we extracted measurements of rod position, orientation, fluorescence and length at evenly spaced timepoints. To simulate noisy measurements, we added Gaussian noise to each of these features, with the noise magnitude based on that observed in real experimental data (Fig 3B, Methods).

Rather than specialising on datasets with specific properties, FAST’s tracking algorithm is designed to be robust to a wide diversity of different conditions by automatically compensating via feature normalization. To test this capability, we varied the parameters of our simulation by adjusting the rod density, self-propulsion force and framerate, as well as the accuracy of feature measurement by adjusting the amount of noise in the measured cell position, length and fluorescent intensity (Table 1). We tested five different values for each of these six parameters, yielding a total of 30 datasets.

**Table 1:** Parameters of the SPR model used to generate synthetic datasets. Here we show both range of values we tested and baseline value that was used when we varied another parameter. Note that our simulations are non-dimensionalised, using the width of a single rod as the characteristic lengthscale and the time taken for an isolated rod with self-propulsion force *ν* = 1 to move a single rod width as the characteristic timescale.

Parameter symbol	Parameter name	Value(s)
***A***	Area of simulation domain	10,000
***f***_**0**_	Stokesian friction coefficient	1
***N***	Number of rods	700 (baseline) [300, 500, 700, 900, 1100] (variable)
***ν***	Self-propulsion force	1 (baseline) [0.7, 0.85, 1, 1.15, 1.3] (variable)
**Δ*T***	Time between sampled timepoints	10 (baseline) [5, 7.5, 10, 12.5, 15] (variable)
***σ***_***r***_	Standard deviation of positional measurement noise	0.02 (baseline) [0.02, 0.1, 0.2, 0.4, 0.6] (variable)
***σ***_***ϕ***_	Standard deviation of orientational measurement noise	0.02 radians
***σ***_***a***_	Standard deviation of length measurement noise	0.1 (baseline) [0.05, 0.1, 0.2, 0.4, 0.6] (variable)
***σ***_***I***_	Standard deviation of fluorescence measurement noise	1 A.U. (baseline) [0.5, 1, 2, 4, 6] A.U. (variable)

The performance of the tracking algorithm was assessed by comparing its output to the ground truth (Fig 3C and 3D). Links that were present in the ground truth but missing in the reconstructions were scored as false negatives (FN), while those that were absent in the ground truth but present in the reconstructions were scored as false positives (FP). Links that were identical in both were scored as true positives (TP). We now integrated these measurements into a single metric that quantifies tracking performance, the *F*_1_-score, defined as:

$$F_{1}=\frac{2\;TP}{2\;TP+FN+FP}.$$ (4)

Users of FAST specify a tracking threshold that controls the stringency of the linking process. If this threshold is too stringent, too many correct links will rejected by the algorithm, while if the threshold is not stringent enough too many incorrect links will be accepted. In practice, users need the ability to choose the tracking threshold that best suits their needs—for example, users interested in detecting very rare events will have different requirements than those interested in measuring average cell behaviour. However, for the purpose of testing the benefits of our automated tracking algorithm, we removed the tracking threshold as a factor from our analyses by using the threshold that resulted in the largest the *F*_1_-score for each dataset (Fig 3E). This maximum *F*_1_-score is an objective measure of the performance of our tracking algorithm across datasets with different characteristics, and also allows us to robustly benchmark our tracking algorithm.

The simulated datasets were tracked using two different methods (Fig 3B). The first method only used the position of the rods—effectively a classical position-based tracking approach (‘Centroids’), while the second method used all four features—position, orientation, length and fluorescent intensity (‘All features’). We found that including all features led to a dramatic increase in tracking accuracy in all datasets, with a 4 to 10-fold reduction in the number of tracking errors (S2 Fig). Furthermore, our groundtruthed datasets allow us to directly relate a dataset’s trackability to the accuracy of the resulting tracks, which demonstrated that it is an excellent predictor of the *F*_1_-score regardless of the simulation parameters or level of noise in the dataset (Fig 3F).

Taken together, these analyses validate our methodology and illustrate how FAST copes with challenging datasets. First, they demonstrate that our method of integrating additional feature information during tracking is reliable and effective, allowing FAST to substantially reduce tracking errors compared to alternative approaches that use cell position alone. Second, these analyses demonstrate that our trackability score can integrate multiple aspects of the dataset into a single, robust heuristic of predicted tracking accuracy.

### Case studies

#### Tracking cells, divisions, and lineages in growing non-motile *E*. *coli* colonies

A major aim of a number many previous tracking packages is to automatically reconstruct cell lineages [25,28]. However, identifying cell division events and the resulting daughter cells is a particularly difficult challenge. Near-perfect tracking of individuals is essential for accurate lineage tracking because single errors can propagate and cause lineages to be misassigned at later timepoints. We tested FAST’s lineage tracking capabilities using eight separate time lapse movies of *E*. *coli* cells as they divided to form microcolonies [11]. These experiments were imaged using a combination of phase-contrast and fluorescence microscopy, and used a strain with a GFP transcriptional reporter to quantify the level of colicin Ib (*cib*) expression. The expression of this gene is highly variable between cells but remains stable over a single generation. This combination of population-level heterogeneity and individual-level stability means that the level of GFP expression in each cell could contribute a large amount of information to FAST’s tracking algorithm and thus substantially improve its performance.

**Fig 4:**
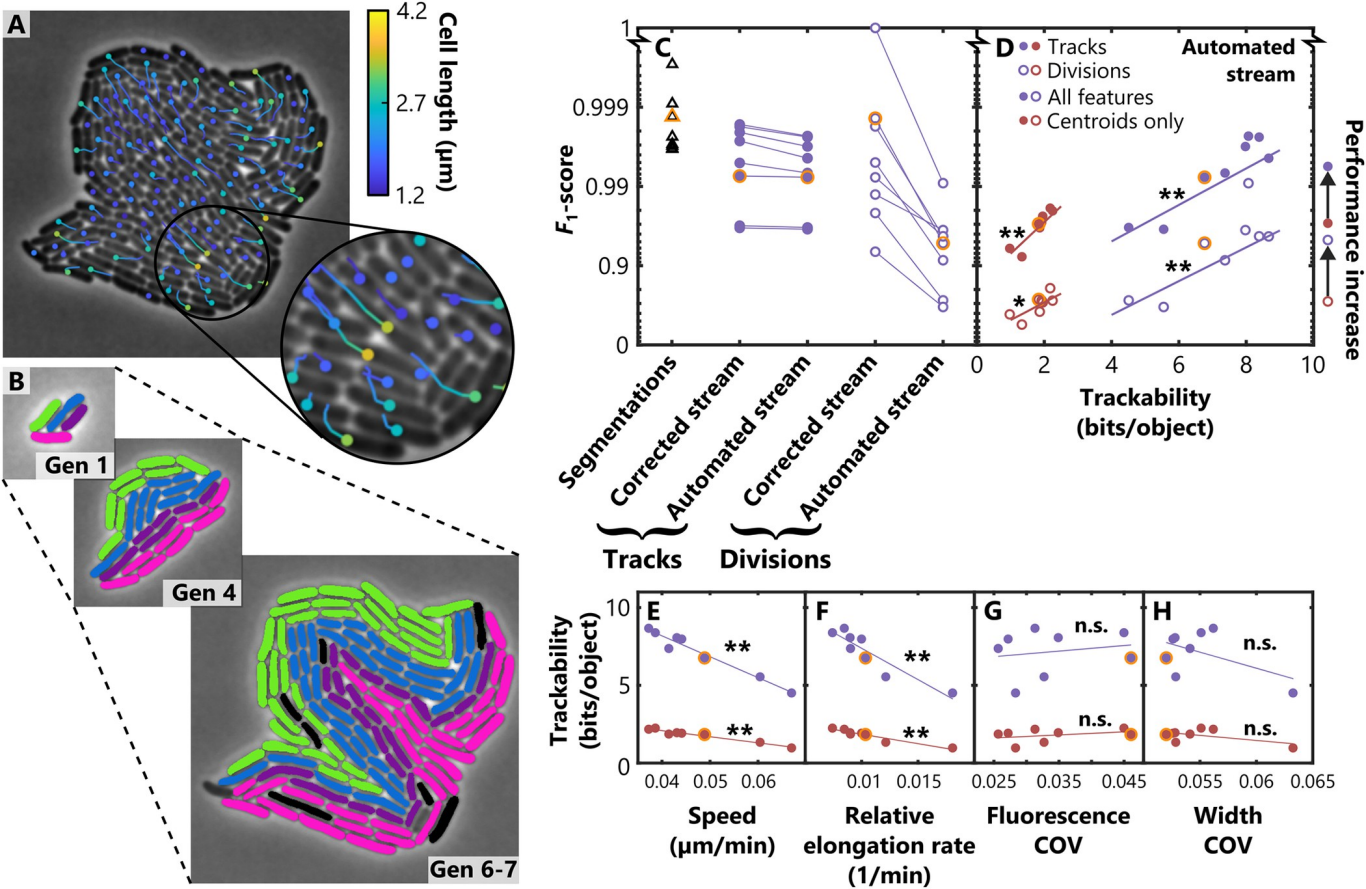
Benchmarking FAST’s performance using an experimental dataset containing eight *E. coli* microcolonies. (A) Tracks showing the movement of individual cells within a typical microcolony as it expands, where colours denote the instantaneous cell length. (B) Automated lineage tracking of microcolony at three different timepoints. Cells that share the same mother in generation 1 (‘Gen 1’) are labelled with the same colour, illustrating how the spatial distribution of different lineages develops over time. Occasional black cells in the final timepoint (‘Gen 6–7’) indicate cells that were not assigned to the correct lineage. (C) Images from eight different microcolonies were processed with FAST to automatically obtain trajectories and division events. These were then compared to a manually curated ground truth to calculate *F*_1_-scores, quantifying the performance of the Segmentation module (triangles), Tracking module (filled circles) and Division Detection module (empty circles). These analyses proceeded in two parallel streams: In the ‘corrected’ stream, the inputs of the Tracking and Division Detection modules had been manually corrected, whereas in the ‘automated’ stream these inputs were obtained directly from the previous module and remained uncorrected. (D) When averaged over all timepoints that contained more than 32 cells, we found that the trackability metric was a reliable predictor of the accuracy of both the Tracking and Division Detection modules under fully automated conditions. In addition, this analysis showed that including cell length, width, fluorescent intensity and position as features in the Tracking module (‘All features’, purple) reduced the median number of errors in both the Tracking and Division Detection modules by approximately 5-fold compared to results obtained using cell position alone (‘Centroids only’, brown). (E-H) We next investigated which factors influence trackability by comparing metrics related to fluctuations in feature values–average cell speed (E), relative elongation rate (the fractional change in cell length per min) (F), coefficient of variation (COV) of GFP intensity (G) and COV of cell width (H). The statistical significance of correlations is denoted using the following symbols: * denotes *p* < 0.05, ** denotes *p* < 0.005, n.s. = not significant. These analyses were performed using the log-transformed *F*_1_-scores in D and are based on a linear regression t-test. The orange points in C-H indicate the exemplar microcolony shown in A and B. Visualisations shown in A and B were produced using FAST’s Overlays module.

We began by applying automated tracking and division detection to a single dataset. We visualised the lineage structure of the microcolony using the Overlays module to rapidly verify the accuracy of the lineage assignment (Fig 4A and 4B, S1 Movie). This revealed that the descendants of each of the individual cells present at the beginning of the experiment formed highly elongated structures within the colony, similar to the patterns previously observed in experiments where colonies were initiated from multiple founder cells labelled with different fluorescent proteins [8]. Next, we used our batch processing tool—doubleFAST—to automate the analysis of the remainder of the eight datasets, using the parameters obtained when analysing the first microcolony. A complete breakdown of processing times by module and dataset is shown in Table 2—in total, processing of all eight datasets took 5.5 minutes, or around 40 seconds per microcolony on a standard laptop computer (Methods).

**Table 2:** Processing times and dimensions of lineage datasets. ‘Dimensions’ indicates the size of the raw imaging dataset as x-size, y-size and duration. x- and y-sizes are recorded in pixels, while duration is recorded as the number of frames. ‘Objects’ indicates the total number of segmented objects in the manually corrected segmentations, while ‘Cells’ indicates the total number of separate cell trajectories in the manually corrected track dataset. The ‘Processing time’ section reports the amount of time taken to process each dataset by each module of FAST (in seconds) on a standard laptop computer (see Methods for details).

				Processing time (s)			
Dataset ID	Dimensions	Objects	Cells	Segmentation	Features	Tracking	Divisions
140408_01_cib	645x634x88	2698	363	26.0	10.2	3.5	16.8
140408_02_cib	600x445x104	1117	164	19.6	5.2	1.0	3.1
140408_09_cib	627x629x76	2398	288	20.8	6.2	2.0	9.9
140408_10_cib	517x831x92	2118	287	27.1	6.5	1.7	5.5
140408_11_cib	517x724x75	1701	198	19.3	4.9	1.9	6.8
140409_03_cib	738x540x82	2968	324	26.9	7.1	2.5	13.0
140415_08_cib	575x670x67	1760	224	24.5	4.9	1.7	9.9
140415_13_cib	489x655x79	2590	301	26.5	5.7	1.9	10.0

Although these automated analyses produced highly accurate results, a few tracking and division assignment errors were observed (Fig 4B). To objectively assess the accuracy of FAST’s algorithms, we benchmarked our results by constructing manually-validated ground-truth versions of the Segmentation, Tracking and Division-Detection module outputs. In the case of the Tracking module, this task was substantially facilitated by our Track Correction GUI. Our analyses then proceeded via two separate streams: in the first, ‘corrected’ stream, we used the manually corrected output of the Segmentation module as an input to the Tracking module, and the manually corrected output of the Tracking module as an input to the Division Detection module, allowing us to assess the quality of each module independently. In the second, ‘automated’ stream, we used the uncorrected outputs of each module as the input to the next. This allows us to assess the performance of the tracking and division detection algorithms in conditions typical of large-scale analyses, where errors from earlier analyses are propagated uncorrected into later steps (Fig 4C).

We found that the results of our Segmentation module were highly accurate, with a median mis-segmentation rate of 0.52%. The Tracking module was similarly accurate, with a median error rate of 1.02% when using the uncorrected segmentations as inputs. This is very similar to the error rate reported for DeLTA 2.0 [36], which was also benchmarked against microcolony datasets from van Vliet et al. [11]. We note, however, that the average error rate reported for DeLTA 2.0 is an aggregate measure that also includes datasets from additional *E*. *coli* genotypes in van Vliet et al. [11] not considered here. Manually correcting FAST’s rare mis-segmentations had a relatively small impact on the Tracking module’s performance, decreasing the median error rate to 0.71%, suggesting that the tracking algorithm’s automated system for bridging mis-segmentations performs as intended. In contrast, the Division Detection module’s performance was considerably enhanced when the input trajectories were manually corrected, with the median error rate decreasing from 4.77% to 0.63%. We therefore recommend using our track correction GUI if highly accurate lineage assignment is required.

We also analysed the utility of trackability as a predictor of tracking algorithm performance, equivalent to our automated benchmarking with the SPR model (Fig 4D). As before, we found that trackability averaged over all timepoints with a relatively large number of cells (>32) was robust predictor of the tracking performance on the different microcolonies. This was even true for individual timepoints, with trackability being a much better predictor of tracking fidelity than more rudimentary metrics such as the current number of cells within the microcolony (S3 Fig). We also found that trackability was a strong predictor of how accurately divisions were detected (Fig 4D). Lastly, we measured the performance of the tracking and division detection algorithms when a large suite of features was used (cell length, width, fluorescence, and position; denoted ‘All features’) compared to when only cell positions were used (denoted ‘Centroids only’). Similar to what was previously observed in the synthetic datasets, adding extra features substantially increased the performance of our algorithms, reducing the number of tracking and division assignment errors by approximately 5-fold.

Given that the eight microcolonies that we analysed used the same strain of bacteria and were grown in the same experimental conditions, we were surprised by the variations in tracking performance that we observed across the eight microcolonies. One potential cause of this variation was that the user-defined tracking parameters which were optimised for one dataset were not optimal for the other microcolonies. However, when we systematically repeated the benchmarks with different user-defined tracking parameters, we found that the set of tracking parameters that produced the most accurate results was highly conserved across the eight microcolonies (S4 Fig), suggesting this was not the primary cause. Next, we asked whether biological differences between the samples could be responsible for the observed variation in performance. To test this, we measured each microcolony’s average cell speed, relative elongation rate (the average fractional increase in cell length per minute), coefficient of variation (COV) of GFP fluorescence and the COV of cell width using our manually corrected tracks, and investigated how these metrics related to the microcolony’s trackability (Fig 4E–4H). We found that the average rate at which cells move and grow within a microcolony were significantly correlated with the microcolony’s trackability. In contrast, the amount of variability observed in the cell fluorescence and cell width did not have an appreciable impact on a microcolony’s trackability. Taken together, these results indicate that the variation in the trackability metric we observed were driven by real biological variation across the different microcolonies. In this case differences in the average growth rate of cells in each microcolony likely drive changes in the speed at which these non-motile colonies expand, and consequently impacts the fidelity of tracking and division detection.

In summary, these analyses show that segmentation errors do not substantially affect the tracking performance of FAST, but errors in tracking can detrimentally impact the detection of cell division events. Moreover, these analyses of experimental data validate our previous findings from the synthetic datasets by demonstrating firstly that tracking performance can be substantially improved by including cell features other than cell position and secondly that trackability is a reliable predictor of tracking fidelity. Finally, this case study demonstrates that the two user-defined tracking parameters *F* and *P* do not need to be excessively fine-tuned to obtain reliable tracking results (S4 Fig, note the logarithmically-spaced axes).

#### Quantifying rapid bursts of cell movement in densely packed *P*. *aeruginosa* monolayers

Many different species of bacteria generate collective motility in densely-packed communities using either flagella, Type IV pili, or via gliding [2,15,35,37]. Motility allows populations to rapidly expand into new territory, giving them a competitive advantage over non-motile genotypes [1,38]. Here we demonstrate how FAST can be used to quantify the behaviour of *P*. *aeruginosa* cells in interstitial colonies that form between agar and glass [37]. We focus on cells within the monolayer that forms directly behind the colony’s leading edge, the dynamics of which play a crucial role in the competition between genotypes in both interstitial and more classical ‘surficial’ colonies [1].

Tracking cells undergoing collective movement requires a much larger acquisition frame rate compared to non-motile cells. While expensive timing boards can be used to ‘trigger’ cameras with a high level of precision to keep the time between frames nearly constant, many high-end research microscopes lack this capability. Instead, ‘camera streaming’ is more widely available, which directly streams the camera’s output to a computer which saves frames as fast as possible. This maximises the frame rate, but images acquired via camera streaming can have slight variations in the time that elapses between subsequent frames.

To illustrate how FAST can be used to handle a sequence of images collected via camera streaming, we collected a large dataset with 3,505 frames recorded at an average rate of 127 frames per second. The size is of this dataset is approximately 8 Gb and each frame contains approximately 1,700 tightly packed *P*. *aeruginosa* cells. Despite the large size of this dataset, processing with FAST could be completed on a standard laptop computer (Methods), requiring only 200 mins to segment and 355 mins to extract the features of each of the ~6 million individual objects.

**Fig 5:**
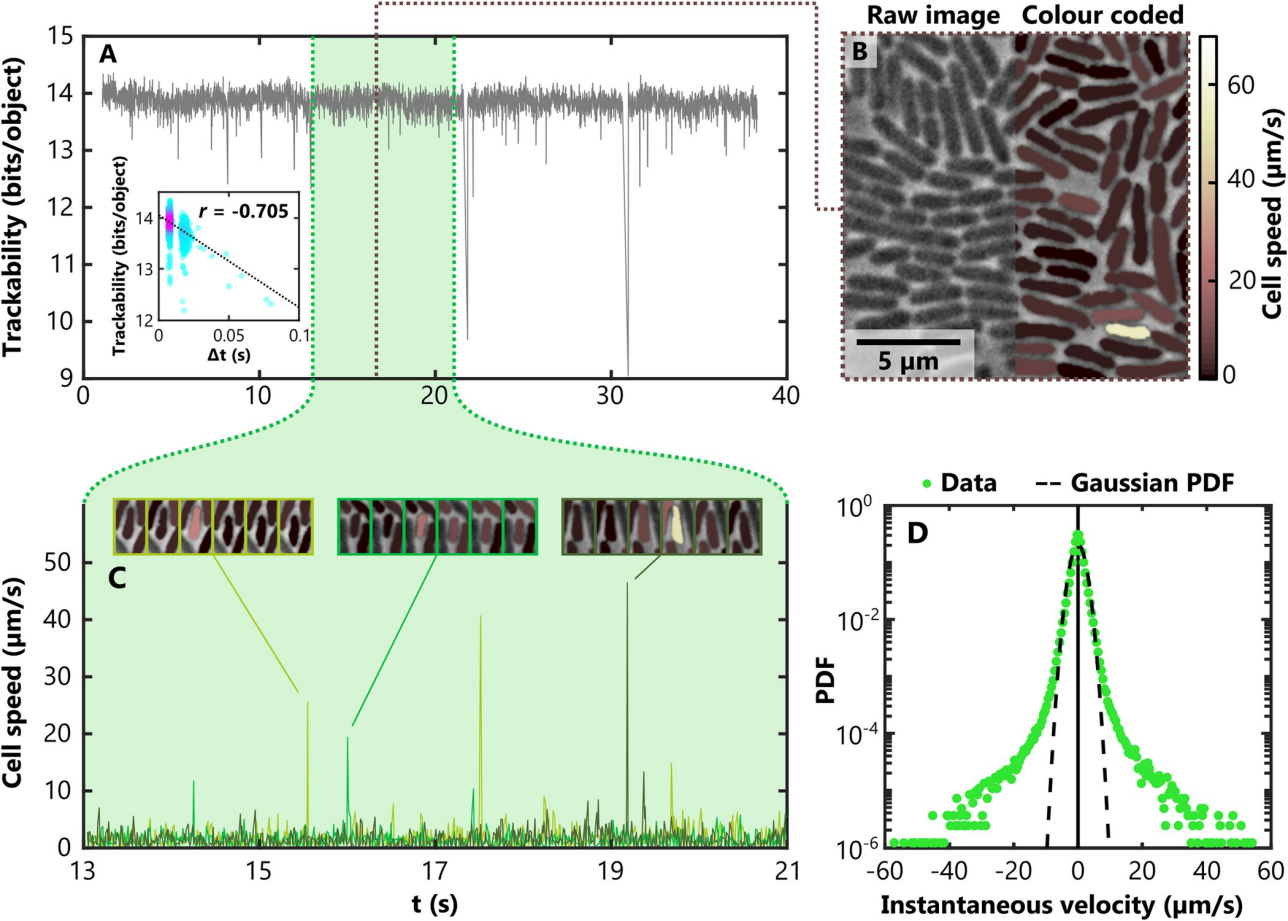
Tracking single cells in an interstitial *P. aeruginosa* colony spreading via pili-based motility. (A) We used FAST to track cells within a monolayer of *P. aeruginosa* undergoing collective motility using position, length and width as features. Images were collected using high speed ‘camera streaming’ with a mean frame rate of 127 fps, but variations in the elapsed time between subsequent frames, *∆t*, resulted in transient reductions in trackability. (A, inset) Analysis of the timestamps associated with each frame revealed that the trackability score was negatively correlated with *∆t* (*r* indicates Pearson’s correlation coefficient; the warmer colours denote a higher density of data points). We therefore restricted our subsequent analyses to a subset of the data in which the trackability score was relatively constant (green region) using FAST’s built-in tools. (B) We used the Overlays module to colour code cells based on their instantaneous speed. Although cells typically moved relatively slowly, very occasionally cells were observed to undergo a very rapid burst of movement (see cream coloured cell). (C) These rapid jumps can also be observed in traces of the speed of individual cells. Here we plot the instantaneous speed of three different cells over time, each in a different colour. The montages above illustrate three of these transient events, using the same colour coding shown in B. (D) To investigate these movements at the population level, we calculated instantaneous cell velocities in both the *x* and *y* direction for all cells and plotted their combined distribution. In other active systems, this distribution is approximately Gaussian. However, in our system the highly transient bursts of velocity result in heavy tails, causing them deviate from a Gaussian distribution with the same variance (dashed black line).

Tracking consists of two separate stages: the model training stage, and the link assignment stage. After completion of the model training stage, FAST automatically generates and plots the trackability of the dataset at each timepoint (Fig 5A). For our dataset, this plot revealed that the trackability dropped precipitously at some timepoints. We hypothesised that these decreases resulted from a reduction in the imaging framerate. To test this, we plotted the trackability score against ∆*t*, the elapsed time between frames as calculated from timestamps (Fig 5A, inset). This revealed a strong negative correlation (Pearson’s correlation coefficient = -0.705), suggesting that the longer the time between frames, the lower the trackability and the less accurate the tracking results. To avoid these timepoints—and the spurious tracking results they might generate—we used the tracking module’s built-in time window selection tool to specify a subset of frames to track (Fig 5A, green region, 750 frames). Training the tracking model for this reduced dataset took 18 minutes, while tracking and track processing took an additional 88 minutes.

In this example, a relatively large timestep ∆*t* allows cells to move further between frames, which reduces predictability and therefore reduces the trackability score. More generally, however, the trackability depends on a combination of both experimental and imaging conditions, providing a robust metric to interpret datasets. For example, the trackability score can be used to quickly identify a wide range of problems which might arise during an experiment, including fluctuations in focus, illumination intensity, or inadvertent movement of the sample. Once problematic timepoints have been identified, the user can decide either to avoid them by using a subset of the images (as in this example) or to reject the entire dataset.

Tracking the smaller subset of images that we specified resulted in over 1,600 separate trajectories, each at least 200 timepoints long. To visualise this large dataset, we used the Overlays module to colour individual cells in the phase-contrast image based on their instantaneous speed (Fig 5B, S2 Movie). This revealed that while most cells move at relatively slow speeds, a small number of cells undergo rapid, sporadic bursts of movement approximately 25 times faster than the average. These rapid movements are also clearly visible in measurements of the speed of individual cells over time (Fig 5C). Our results are similar to the ‘slingshots’ previously observed in solitary *P*. *aeruginosa* cells moving at the glass/liquid interface, apparently driven by pilus detachment events [39]. However, the peak speeds that we record (up to 60 μm s^-1^) in collectives of *P*. *aeruginosa* are approximately 20 times larger than those previously observed. While we do not know the specific reason for this difference, interactions with neighbouring cells facilitated by the high cell density, the glass/agar interstitial colony environment and our higher framerate (~13 times that of [39]) may each play a role.

To illustrate how this large tracking dataset can be mined to elucidate the statistics of rare events, we constructed the instantaneous marginal velocity distribution from our tracks (*i*.*e*. the *x*- and *y*-components of the instantaneous velocity vectors) (Fig 5D). While previous studies have found that sperm and swimming bacterial cells undergoing collective movement generate marginal velocity distributions that are approximately Gaussian [35,40], we observed that bacteria collectively moving via pili-based motility exhibit much heavier tails corresponding to their occasional rapid motions. Despite the rarity of these events—less than 0.05% of our measurements had a magnitude larger than 20 μm s^-1^—the exceptional number of cell trajectories we obtained nevertheless allowed us to finely resolve their statistical distribution. These analyses demonstrate how FAST can be used to rapidly characterise the motility of a large number of cells in densely-packed conditions, with relatively little user input and computational effort.

#### Automated analysis of T6SS battles

To illustrate FAST’s capabilities beyond standard cell tracking, we analysed the activity of the Type 6 Secretion System (T6SS) using FAST. The T6SS is a highly dynamic organelle composed of a molecular ‘spear’ tipped with a toxin known as an effector [41]. Bacteria that possess the T6SS can inject this effector into neighbouring cells, which kills them [42]. Firing events can be monitored by visualising the localisation of the protein that forms the contractile sheath of the T6SS needle, TssB in *P*. *aeruginosa* and VipA in *Vibrio cholerae* [12,43]. Here, we highlight how FAST can be used to quantify how the T6SS is regulated in co-cultures of *V*. *cholerae* and *P*. *aeruginosa*.

**Fig 6:**
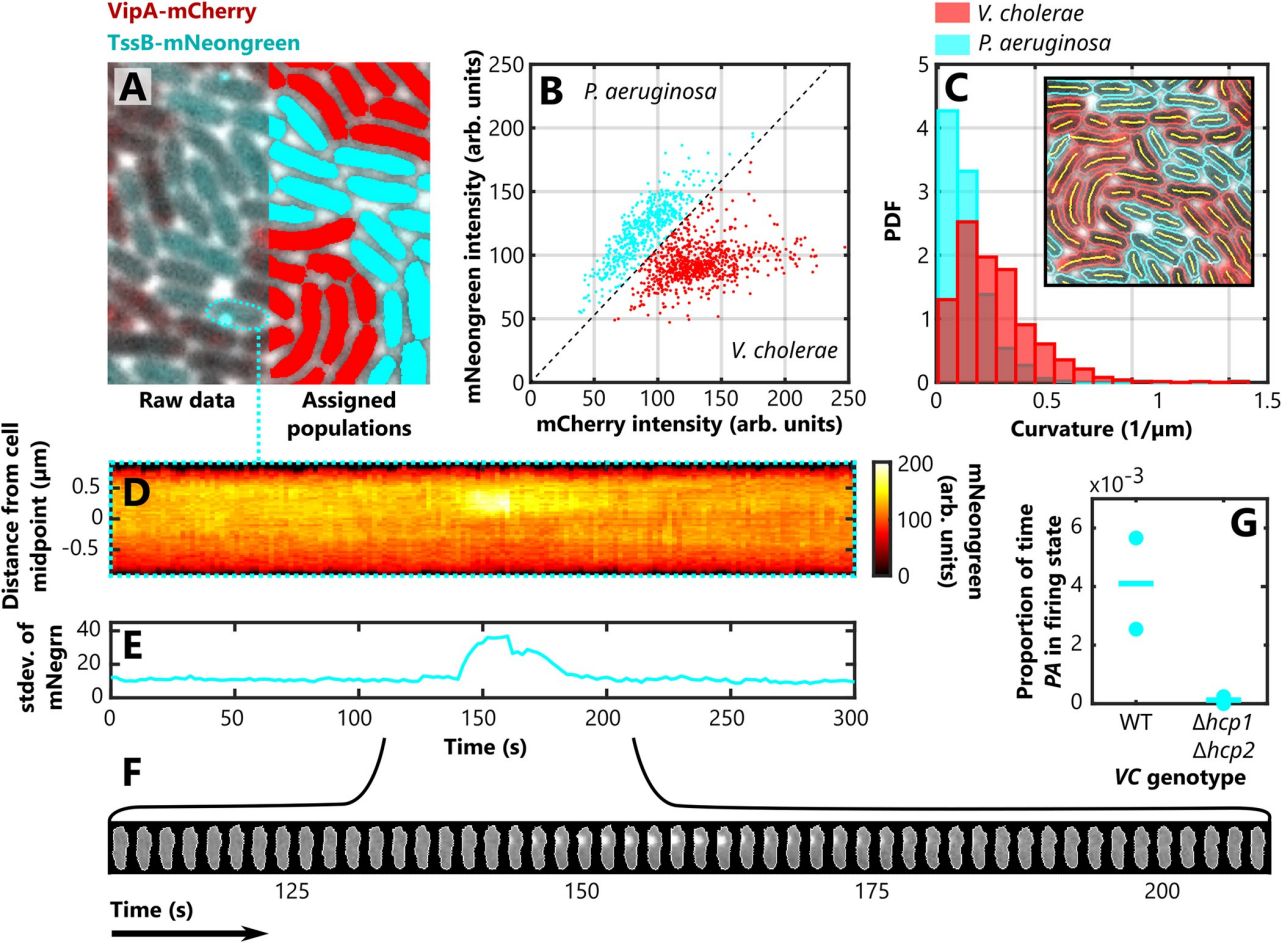
Identifying different bacterial species, quantifying cell morphologies, and investigating T6SS dynamics in a densely packed collective. (A) A monolayer composed of a mixture of *P. aeruginosa* cells expressing TssB-mNeongreen and *V. cholerae* cells expressing VipA-mCherry (left). Measurements of the average intensity of each cell in the two different fluorescence channels allowed us to automatically distinguish the two species (B), an approach known as image cytometry. By colour coding each cell (*P. aeruginosa*, cyan, *V. cholerae*, red), we were able to visually confirm the accuracy of the assignment process (A, right). (C) We then quantified a novel feature, cell curvature, using FAST’s custom feature extraction framework to calculate the curvature of the segmentation backbones (inset). Splitting these measurements into the two previously assigned populations showed the comma shaped *V. cholerae* cells were substantially more curved than the rod-shaped *P. aeruginosa* cells. (D-F) We then used FAST’s plotting options to illustrate the dynamics of the T6SS. A *P. aeruginosa* cell fires its T6SS machinery (observed as the bright puncta of TssB), which can be visualised using the kymograph (D) and ‘cartouche’ (F) plotting options, and which also leads to an increase in the standard deviation of the mNeongreen channel (E). (G) We used this latter measurement to detect when the T6SS was active in a cell, allowing us to measure the proportion of time that each cell in the population spends in the firing state. Our analyses confirmed that when co-cultured with a strain of *V. cholerae* with an inactive T6SS (Δ*hcp1* Δ*hcp2*), the T6SS activity of WT *P. aeruginosa* cells is dramatically reduced compared to when they are co-cultured with WT *V. cholerae*. Here the circles show averages from separate movies and horizontal lines show the mean for all samples of a given combination of genotypes.

Because of its short range, the T6SS is effective only at high cell density, which historically has made it difficult to study using automated analyses. We used FAST to analyse imaging datasets that show *V*. *cholerae* and *P*. *aeruginosa* cells expressing VipA-mCherry and TssB-mNeongreen, respectively, interacting through their respective T6SSs when mixed together in a densely-packed monolayer [12] (Fig 6A, left). We distinguished the two species from one another (Fig 6A, right) using FAST’s post-processing toolbox, which compares each cell’s intensity in the mCherry and mNeonGreen channels to automatically assign them to two distinct populations (Fig 6B). This utility allows one to rapidly compare the behaviour of different genotypes within the same sample.

The architecture of FAST allows users to easily define and quantify novel features that are not already included in FAST. To do this, users can write a short feature measurement script that can make use of the segmentation of each object in each frame, as well as the corresponding regions in each of the imaging channels—in this case, the mNeongreen, mCherry and phase-contrast signals. The Feature Extraction module then automatically stores these custom features in the same way as the built-in features, allowing them to be integrated into downstream analyses. To illustrate this capability, we extracted cell curvature as a custom feature to see if we could detect the differences in morphology of *V*. *cholerae* cells (which are comma shaped) from that of *P*. *aeruginosa* (which are rod shaped). Using a skeletonization-based approach, we extracted the morphological backbone of each cell from its segmentation and measured its curvature by finding the best-fit circle to this set of points. Consistent with expectations, our automated analysis found that the cells identified as *V*. *cholerae* were substantially more curved than those identified as *P*. *aeruginosa* (Fig 6C).

Our software is also capable of generating sophisticated visualisations and analyses of the behaviour of single cells. For example, FAST allows the dynamics of T6SS firing in individual cells to be easily visualised using the kymograph option of the Plotting module (Fig 6D), as well as the ‘cartouche’ option, which extracts and aligns cropped images of the specified cells (Fig 6F). We can also quantify the dynamics of the T6SS using the feature data associated with a given cell: the standard deviation of the mNeongreen channel substantially increases during firing (Fig 6E), as the TssB becomes non-uniformly distributed through the cell as it assembles into the sheath.

It is generally accepted that *P*. *aeruginosa* only fires its T6SS when triggered by the firing of the T6SS of a neighbouring cell [9,12]. Although qualitative evidence for this is strong, to our knowledge this effect has never been directly quantified, likely because of the difficulties involved with tracking densely packed cells and because the T6SS firing events are themselves relatively rare. We therefore decided to use FAST to simultaneously track a large number of densely packed cells and automatically detect when each fires its T6SS. We used doubleFAST to automate the analysis of multiple datasets from experiments in which WT *P*. *aeruginosa* cells were either co-cultured with WT *V*. *cholerae* or co-cultured with a mutant *V*. *cholerae* strain that is incapable of firing their T6SS (Δ*hcp1* Δ*hcp2*). Using the standard deviation of the mNeongreen channel to distinguish when *P*. *aeruginosa* is actively firing its T6SS, we calculated the proportion of time that *P*. *aeruginosa* cells spend firing their T6SS (Fig 6G, S3 Movie). As expected, when co-cultured with the inactivated Δ*hcp1* Δ*hcp2 V*. *cholerae* strain, *P*. *aeruginosa* dramatically reduced the proportion of time it spent firing its T6SS compared to when co-cultured with WT *V*. *cholerae*. These analyses generated 1,246 separate trajectories, of which around half (601) spanned the full 151 timepoints of the experiments. In conclusion, this case study demonstrates how FAST can automatically distinguish different species, how users can specify novel features like cell curvature, and how complex bacterial behaviours can be quantified using feature data.

## Discussion

Advances in experimental techniques and automated microscopy have transformed live cell imaging from a technique that relies largely on qualitative observation into a highly quantitative discipline that leverages huge amounts of data. Integral to this renaissance are computational tools that can automatically parse and annotate the large imaging datasets resulting from these experiments. Many tools have been developed for this purpose [17,24–28,44–47], each optimised for a specific research question. As we have emphasized throughout this manuscript, our own contribution is particularly well-suited for the analysis of experiments where cells are tightly packed together.

We designed FAST to allow users to rapidly obtain high-quality results by minimising both the number of user-defined parameters in the Tracking module and the computational time required to process an imaging dataset containing many cells. Our tracking algorithm automatically determines how to combine data from different cell features, which prevents users from having to iteratively improve tracking results by sequentially adjusting a large number of parameters—typically a very slow and laborious process. In the case of the microcolony analyses of Fig 4, selecting appropriate values of the user-defined tracking parameters *F* and *P* took only a few minutes using FAST’s built-in tools. Subsequent benchmarking showed that the specific set of *F* and *P* parameters chosen for the first microcolony were nearly optimal for all eight datasets (S4 Fig). This robustness allows users to queue the autonomous analysis of many datasets using doubleFAST. Our approach also provides the user with a single, easy to interpret metric that allows them to rapidly identify and avoid sections of datasets that are predicted to yield low-fidelity trajectories. While the basic FAST package already outputs most of the cell features that are widely used by researchers, simple modifications to the system allow users to integrate new features into the existing analytical framework.

We also note some limitations to our approach. Firstly, large-scale shifts in field of view, for example caused by thermal drift or the imprecise movement of a motorised stage, can adversely impact the tracking accuracy of FAST. While the timepoints at which these shifts occur can be pinpointed by the associated sharp reductions in trackability (and potentially excluded from subsequent analyses, similar to the process shown in Fig 5A), it is usually better to stabilise troublesome datasets with tools such as TurboReg [48] before processing them with FAST. We also note that the reliance of our approach on summary statistics such as Σ_*t*_(Δ***x***) (the covariance matrix of feature displacements) means that it can suffer from stability issues when the number of cells in the field of view is very low (≲10), as there is insufficient training data to accurately estimate these statistics. However, should this pose an issue—for example at the beginning of microcolony datasets when there are only small numbers of cells—the relatively small amounts of data involved generally make it feasible to correct these errors using the manual track correction GUI.

Tracking systems typically break down in one of two ways—either individual objects cannot be distinguished, or the tracking algorithm cannot accurately link individuals between frames. Machine learning is increasingly used to solve the first of these two challenges, and a number of software packages which use deep learning methods to segment microbes at high density are now widely available [36,46,47,49,50]. However, the application of such techniques to the tracking problem has gained traction only in the last few years [36,51–53]. The approach described here is based on a statistical framework that optimises track quality while simultaneously providing a metric that predicts trajectory reliability. Our software has already proven its ability to elucidate the rich and complex behaviours that individual bacteria exhibit in densely-packed conditions [1,10,22,23]. Future work stemming from these combined experimental and analytical approaches could ultimately shed new light on how dense bacterial communities function and help us to develop novel strategies to manipulate them.

## Online methods

### Computational resources

All analyses presented in this manuscript were performed with a Microsoft Surface Book 2, with an 8-core Intel i7-8650U CPU and 8 Gb of RAM. FAST was run directly in Matlab, version 2018b.

### SPR model

The 2D SPR model used in this manuscript has been described in detail elsewhere [1,35]. In brief, we model cells as stiff rods composed of evenly-spaced Yukawa segments, mutually repulsive point potentials. We initialise the system by filling a square domain with *N*_*r*_ rods, evenly spaced on a lattice. Each rod *i* is associated with an aspect ratio *a*^*i*^ and a fluorescence intensity *I*^*i*^, randomly drawn from distributions fitted to *P*. *aeruginosa* data from an experiment visualising the T6SS [9] as shown in S1 Fig. The packing fraction of the system, *ρ*, is calculated as

$$\rho =\frac{1}{A}\sum_{i=1}^{N_{r}}\left[(a^{i}-1)+\frac{\pi }{4}\right],$$

where *A* is the area of the simulated domain, which has doubly periodic boundary conditions.

Taking the instantaneous position of a rod *i* as ***r***^*i*^, its orientation as *ϕ*^*i*^, the unit vector denoting this orientation as ${\hat{u}}^{i}$ and the sum of the potentials between *i* and all other rods as *U*^*i*^, we define the equations of motion for each rod as:

$$f_{T}∙\frac{\partial r^{i}}{\partial t}=-\frac{\partial U^{i}}{\partial r^{i}}+\nu {\hat{u}}^{i},$$ (5A)

$$f_{\phi }\frac{\partial \phi^{i}}{\partial t}=-\frac{\partial U^{i}}{\partial \phi^{i}},$$ (5B)

where ***f***_*T*_ is the translational friction tensor, *f*_*θ*_ is the rotational friction constant and *ν* is the size of a self-propulsion force exerted by each rod along its axis. We use the formulation presented in [35] to calculate ***f***_*T*_ and *f*_*ϕ*_, which are in turn functions of the rod aspect ratio and the Stokesian friction coefficient, *f*_0_. We simulate the dynamics of our system by numerically integrating Eqs 5A and 5B using the midpoint method.

Following an initial transient, the system reaches a statistical steady-state. At this time, we begin to measure the positions, orientations, lengths and intensities of each rod at a sampling “framerate” Δ*T*. We add simulated measurement noise to each of these measurements, which is drawn from a Gaussian distribution with a mean of zero. The standard deviation of the measurement noise for each feature is controlled by the parameters *σ*_*r*_ (positional noise), *σ*_*ϕ*_ (orientational noise), *σ*_*a*_ (length noise) and *σ*_*I*_ (fluorescence noise). To constrain the baseline estimates of these noise parameters, we measured how each of the corresponding features fluctuated about the mean in the T6SS firing dataset [9]. The cells in this dataset are non-motile and the framerate is high enough that growth is negligible, meaning any apparent changes in position, orientation, length or fluorescence are wholly attributable to measurement noise.

We varied the properties of our simulations by adjusting the values of the parameters *N*, *v*, Δ*T*, *σ*_*r*_, *σ*_*a*_ and *σ*_*I*_ in different simulation runs. The values of these parameters, as well as the fixed system parameters, are provided in Table 1.

### Sample preparation

The monolayer of *P*. *aeruginosa* presented in Fig 5 was prepared using the wild-type PAO1 strain [54]. Cells were streaked out from freezer stocks onto LB agar plates (Lennox, 20 g/l, Fisher Scientific, solidified with 1.5% (w/v) agar, Difco brand, BD) and incubated overnight at 37°C. Single colonies were picked from the resulting plates and incubated overnight in liquid culture under continuous shaking, resulting in stationary phase cultures. These were then diluted 30-fold and returned to the shaking incubator for a further two hours, yielding cultures in exponential phase. The final culture used for inoculation was prepared by adjusting the optical density at 600 nm (OD_600_) of the exponential phase cultures to 0.05 using fresh LB, corresponding to an approximate concentration of 12,500 cells μl^-1^_._

We prepared monolayers from these cultures using a similar protocol to that described in [37]. 1 μl of inoculation culture was spotted onto the centre of a small (2 cm x 2 cm) LB agar pad. To provide optimal conditions for observing twitching motility, the concentration of agar in these pads was 0.8%. This pad was then inverted and placed into the base of a coverslip-bottomed Petri dish (175 μm coverslip thickness, MatTek), which was then closed and incubated for 16 hr at room temperature. The ability to close the lid of the Petri dish allowed us to avoid desiccation of the sample during incubation. By the end of the incubation period, a large interstitial colony with a dense monolayer at its perimeter had formed [1].

### Microscopy

The high framerate movie used for the analysis of rapid motion (Fig 5) was acquired using a Zeiss Axio Observer.Z1 microscope outfitted with an Axiocam 702 camera set to ‘camera streaming’ mode and a Plan Apochromat 63x oil-immersion objective.

### Other datasets

The datasets of dividing *E*. *coli* cells (Fig 4) were downloaded from the raw data repository associated with reference [11], https://zenodo.org/record/268921. They were then stabilised using a customised version of the TurboReg plugin [48]. The T6SS firing dataset (Fig 6) was downloaded from the Supplementary Information section of reference [12] under a Creative Commons Attribution license. FIJI’s [55] built-in AVI reader was used to extract individual frames of this movie.

## Supporting information

S1 Fig Distributions used to specify initial conditions of the SPR model. The distributions of trajectory-averaged aspect ratios (A) and GFP intensities (B) of a dataset of non-motile *P*. *aeruginosa* cells [9]. A gamma distribution (shape parameter = 15.3, scale parameter = 0.248) and a normal distribution (mean = 63.1, standard deviation = 8.83), respectively, were fitted to these two datasets (black dotted lines). To initialise the SPR model, rod aspect ratio, *a*^*i*^, and simulated fluorescence intensity, *I*^*i*^, were randomly drawn from these two fitted distributions, allowing us to ensure that these two features were modelled realistically in our simulations.
(TIFF)

S2 Fig Including additional feature information improves trackability and increases tracking fidelity. We measured the trackability (A, B) and maximum *F*_1_-scores (C, D) of synthetic high-density motility data generated using a range of different parameter combinations. Both ‘simulation parameters’ (parameters that change the properties of the SPR model we used to generate the synthetic dataset, A, C) and ‘measurement parameters’ (parameters that change the amount of measurement noise for each of the different features, B, D) were varied. See Table 1 for further details. Here we compare trackability and tracking fidelity when the tracking algorithm can only use positional information (‘Centroids’, brown) to when all feature information is available (‘All features’, purple). Arrows show the consistent increase in these two metrics when all features from the synthetic dataset are used, illustrating the robustness of our approach.
(TIFF)

S3 Fig The trackability metric is a more reliable predictor of tracking performance compared to the number of cells within a microcolony. We used manually corrected ground-truth datasets to calculate the performance of FAST’s tracking algorithm at each time point of the microcolony datasets shown in Fig 4, using either the full suite of features (purple) or only the cell positions (brown). For all time points that contained 32 or more cells, we then compared the resulting *F*_1_-scores to (A) the instantaneous trackability and (B) the number of cells in the microcolony. For all four regressions, the correlation between the predictor and the *F*_1_-score was significant (*p* < 0.05, linear regression t-test), however the strength of the correlation was much higher for trackability than cell number, as indicated by the corresponding Pearson correlation coefficients (*r*).
(TIFF)

S4 Fig Cell tracking is robust to changes in the two user-defined parameters and produces consistent results across experimental datasets. We tracked cells in each of the eight *E*. *coli* microcolonies forty-nine different times, each time with a different set of the two user-defined parameters—*F*, the proportion of links included in the training dataset and *P*, the tracking threshold. These two parameters allow the user to balance the trade-off between trajectory quality and trajectory quantity during the initial training stage and the primary tracking stage, respectively. We then compared the results of these automated analyses with a manually curated ground-truth to calculate the *F*_1_–score, which measures overall tracking fidelity. These analyses were repeating using both the full set of features (length, fluorescent intensity, width and position, above) and only the cell positions (below). In both cases, we found a wide basin of [*F*, *P*] parameter values that produced a similar level of tracking performance. Moreover, the combination of [*F*, *P*] values that produced the best results was similar between the different microcolonies, suggesting that a set of [*F*, *P*] optimised for one set of experimental images can be applied to subsequent datasets without adversely affecting tracking performance (note the logarithmic axes). The values of *F* and *P* used in the analyses of Fig 4 are shown with black squares.
(TIFF)

S1 Movie Tracking cells, divisions, and lineages in a growing *E*. *coli* microcolony. Growth of *E*. *coli* cells in a microcolony was imaged using phase contrast and fluorescence microscopy [11] (left). The left-hand panel shows a combination of the original phase contrast and fluorescence images, the middle panel shows the individual cell trajectories (where colours denote the instantaneous cell length), and the right-hand panel shows the “lineage tree”. In the lineage tree, the thin lines denote the trajectories of individual cells and thick lines represent division events—both are colour-coded to denote the number of generations elapsed from the original founder cell. This movie shows the same ‘automated’ analysis presented in Fig 4 A and B, in which nothing has been manually corrected. All graphics were generated by FAST’s built in Overlays module. The image datasets were obtained from a previous study [11]. Shown here is Dataset ID 140408_01_cib (see Table 2). Elapsed time: 7 hours 5 minutes.
(AVI)

S2 Movie Quantifying rapid, slingshot-like movements of single *P*. *aeruginosa* cells in a densely-packed monolayer. Cell segmentations are coloured-coded (using the Overlays module) by their instantaneous speed, ranging from stationary (black) to the maximum detected cell speed (light orange). Circles show time points interpolated by the tracking algorithm when cells were temporarily mis-segmented. Here we only show the trajectories longer than 200 frames that were included in our final analyses. While FAST outputs many shorter trajectories, these were generally terminated due to multiple consecutive cell mis-segmentations. Thus, to be conservative we only used very long trajectories to quantify the rare slingshotting events (Fig 5D). This movie shows a 230 frame subset of the entire 750 frame long dataset. Elapsed time: 1.809 seconds.
(AVI)

S3 Movie Automated detection of *P*. *aeruginosa* T6SS firing events. We automatically detect firing events of the Type 6 Secretion System (T6SS) using a function from the FAST toolbox that autonomously identifies ephemeral increases in the standard deviation of the GFP pixel intensity within a cell’s segmentation (Fig 6E), which result from the assembly and contraction of the fluorescently labelled sheath that surrounds the T6SS needle. The firing events are marked by red circles (using the Overlays module), which are centred on the cell centroid and scaled by the cell length, over greyscale images showing GFP intensity. These imaging datasets were obtained from a previous study [12]. Elapsed time: 3 minutes.
(AVI)

S1 Text Design goals and module specifications. Includes Fig A-E.
(PDF)
