# Supplementary figures and images for: Tracking bacteria at high density with FAST, the Feature-Assisted Segmenter/Tracker
Source: PLoS Comput Biol. 2023 Oct 9;19(10):e1011524. doi: 10.1371/journal.pcbi.1011524 (PMC10586697; doi:10.1371/journal.pcbi.1011524)

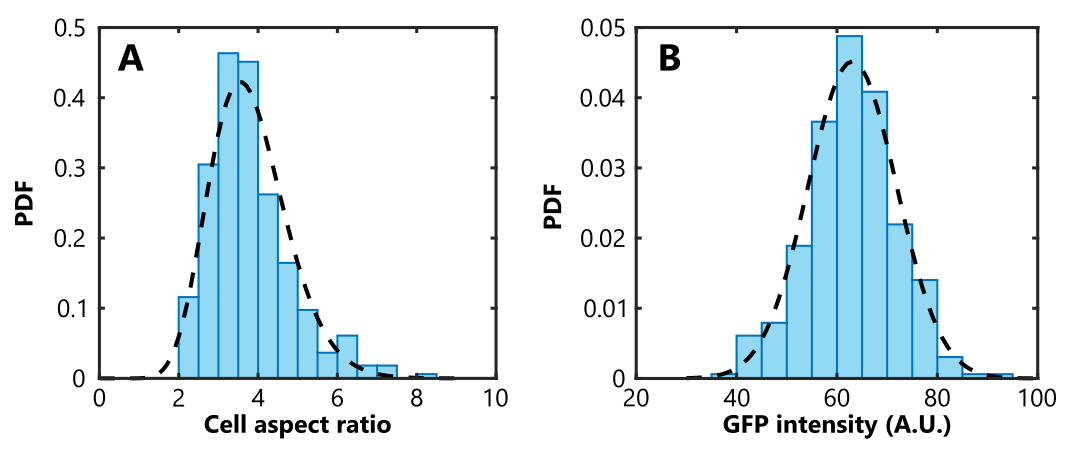

Supplement: S1 Fig — The distributions of trajectory-averaged aspect ratios (A) and GFP intensities (B) of a dataset of non-motile P. aeruginosa cells [9]. A gamma distribution (shape parameter = 15.3, scale parameter = 0.248) and a normal distribution (mean = 63.1, standard deviation = 8.83), respectively, were fitted to these two datasets (black dotted lines). To initialise the SPR model, rod aspect ratio, ai, and simulated fluorescence intensity, Ii, were randomly drawn from these two fitted distributions, allowing us to ensure that these two features were modelled realistically in our simulations. (TIFF) [file pcbi.1011524.s001.tiff]

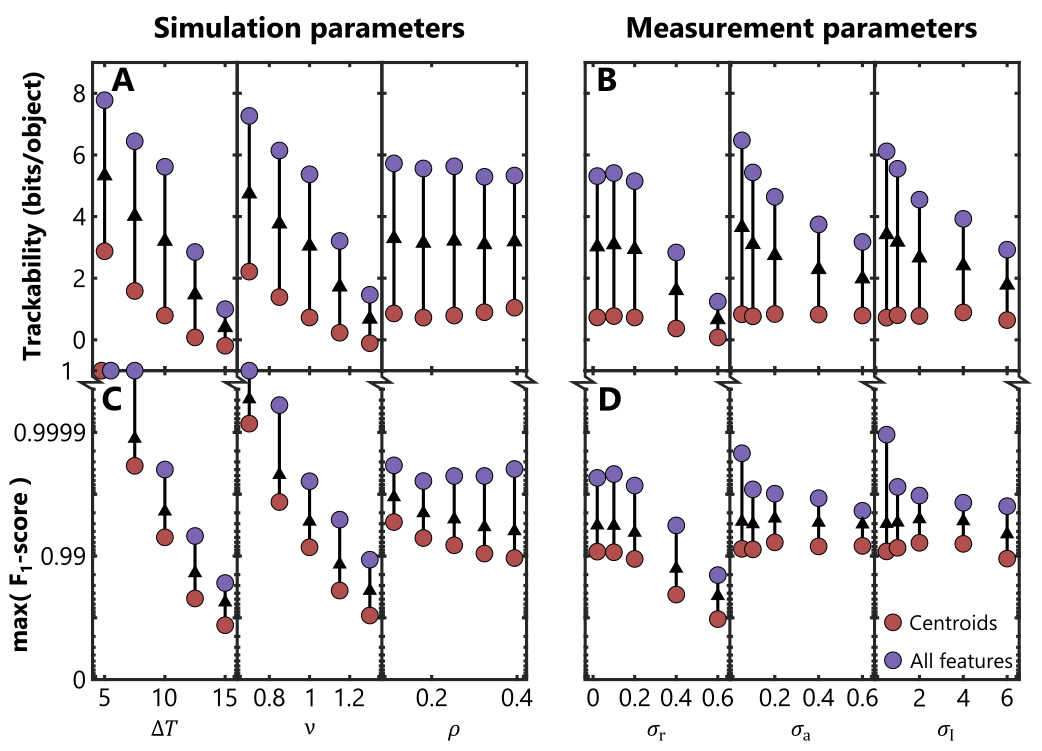

Supplement: S2 Fig — We measured the trackability (A, B) and maximum F1-scores (C, D) of synthetic high-density motility data generated using a range of different parameter combinations. Both ‘simulation parameters’ (parameters that change the properties of the SPR model we used to generate the synthetic dataset, A, C) and ‘measurement parameters’ (parameters that change the amount of measurement noise for each of the different features, B, D) were varied. See Table 1 for further details. Here we compare trackability and tracking fidelity when the tracking algorithm can only use positional information (‘Centroids’, brown) to when all feature information is available (‘All features’, purple). Arrows show the consistent increase in these two metrics when all features from the synthetic dataset are used, illustrating the robustness of our approach. (TIFF) [file pcbi.1011524.s002.tiff]

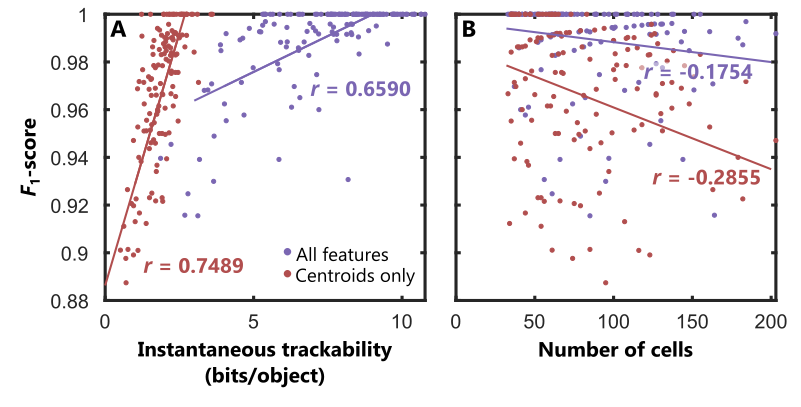

Supplement: S3 Fig — We used manually corrected ground-truth datasets to calculate the performance of FAST’s tracking algorithm at each time point of the microcolony datasets shown in Fig 4, using either the full suite of features (purple) or only the cell positions (brown). For all time points that contained 32 or more cells, we then compared the resulting F1-scores to (A) the instantaneous trackability and (B) the number of cells in the microcolony. For all four regressions, the correlation between the predictor and the F1-score was significant (p < 0.05, linear regression t-test), however the strength of the correlation was much higher for trackability than cell number, as indicated by the corresponding Pearson correlation coefficients (r). (TIFF) [file pcbi.1011524.s003.tiff]

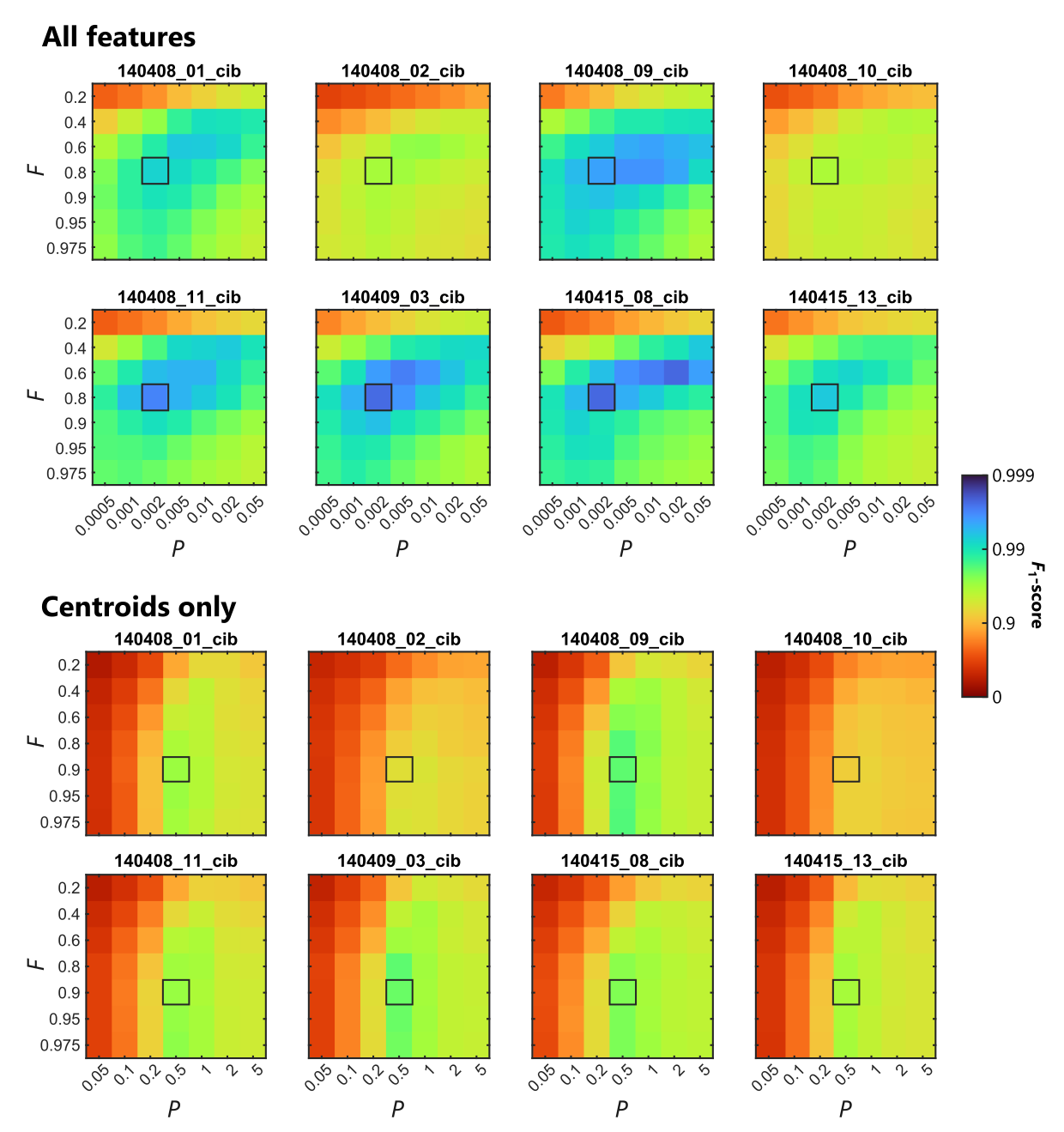

Supplement: S4 Fig — We tracked cells in each of the eight E. coli microcolonies forty-nine different times, each time with a different set of the two user-defined parameters—F, the proportion of links included in the training dataset and P, the tracking threshold. These two parameters allow the user to balance the trade-off between trajectory quality and trajectory quantity during the initial training stage and the primary tracking stage, respectively. We then compared the results of these automated analyses with a manually curated ground-truth to calculate the F1–score, which measures overall tracking fidelity. These analyses were repeating using both the full set of features (length, fluorescent intensity, width and position, above) and only the cell positions (below). In both cases, we found a wide basin of [F, P] parameter values that produced a similar level of tracking performance. Moreover, the combination of [F, P] values that produced the best results was similar between the different microcolonies, suggesting that a set of [F, P] optimised for one set of experimental images can be applied to subsequent datasets without adversely affecting tracking performance (note the logarithmic axes). The values of F and P used in the analyses of Fig 4 are shown with black squares. (TIFF) [file pcbi.1011524.s004.tiff]
